# Markers of left atrial cardiopathy and cognitive function trajectories in adults aged ≥45 years without atrial fibrillation: a population-based study

**DOI:** 10.21203/rs.3.rs-6872566/v1

**Published:** 2025-08-19

**Authors:** Zhe Li, Danielle Marion, Jessica Blair, Elsayed Z. Soliman, David Gladstone, Hooman Kamel, David Birnie, Doug Manuel, Frederick W. Unverzagt, Virginia Howard, Jodi D. Edwards

**Affiliations:** University of Ottawa Heart Institute; University of Ottawa Heart Institute; The University of Alabama at Birmingham; Wake Forest School of Medicine; ICES Central; Weill Cornell Medicine; University of Ottawa Heart Institute; University of Ottawa; Indiana University; The University of Alabama at Birmingham; University of Ottawa Heart Institute

**Keywords:** cognitive trajectory, atrial cardiopathy, biomarkers, atrial fibrillation

## Abstract

**Introduction:**

The study aims to investigate associations between markers of atrial cardiopathy and cognitive function trajectories in adults without prior AF.

**Methods:**

The study included 4,486 participants from the REasons for Geographic and Racial Differences in Stroke (REGARDS) study. Markers of atrial cardiopathy included atrial premature complexes and N-Terminal pro-Brain Natriuretic Peptide (NT pro-BNP). Cognitive status was measured using the Six-Item Screener (SIS). Cognitive status trajectories were identified using latent class growth models.

**Results:**

Three unique cognitive trajectories over the 8-year follow-up were identified: 86.7% showed a normal and stable trajectory of SIS score, 7.6% showed a progressively decreasing trajectory, and 5.7% showed a dramatic decreasing trajectory. Age, black participants, and antidepression drugs were significantly associated with the “dramatic decrease” trajectory. Elevated NT pro-BNP was significantly associated with the “dramatic decrease” trajectory.

**Discussion:**

Markers of left atrial cardiopathy may have significant implications for early diagnosis and prevention of cognitive impairment.

## BACKGROUND

Dementia is a growing epidemic among aging adults, affecting approximately 55 million globally.^1,2^ Dementia is a leading cause of mortality and disability and has a dramatic effect on the healthcare system and resources.^2,3,4^ Early intervention for dementia can delay functional decline and institutionalization, emphasizing the critical need for reliable predictors of dementia risk in the aging population.^5^ Atrial fibrillation (AF) is the most common cardiac arrhythmia, independently increasing the risk of stroke and cognitive.^6,7,8,9^ If detected, the risk of AF-related stroke and dementia can be reduced significantly with anticoagulation treatment.^10^ However, AF is often paroxysmal or clinically silent, potentially limiting the utility of basing risk prediction for dementia on AF.^11,12^

Clinical AF is often preceded by atrial cardiopathy, a series of structural abnormalities and electrical left atrial remodeling.^13^ There is growing evidence that both AF and stroke are potential results of an underlying atrial cardiopathy, suggesting that atrial cardiopathy may be an important risk marker to identify individuals at risk of AF and stroke.^14^ Several studies have shown that markers of atrial cardiopathy, including increased P-wave terminal in lead V1, left atrial enlargement, and premature complexes, increase the risk of AF and stroke.^15,16,17,18,19^ Further, prior work from our team has also demonstrated that adding markers of atrial cardiopathy to existing stroke prediction tools (CHA_2_DS_2_-VASc) improves the predictive utility of the CHA_2_DS_2_-VASc score for stroke risk in those without AF.^20^ Emerging data have shown that atrial structure may also show associations with cognitive decline and dementia in those with AF^9,21^; however, data on whether atrial cardiopathy are associated with cognition in those with no documented AF remains limited.^22,23^ The present study aims to investigate associations between atrial cardiopathy and cognitive function trajectories in aging adults without prior AF. Specifically, this study used data from a population-based cohort study to identify unique cognitive function trajectories in those with measures of atrial premature complexes and N-Terminal pro-Brain Natriuretic Peptide (NT pro-BNP). Atrial premature complexes are a type of arrythmia characterized by premature heartbeats within the atria and have been shown to be a marker of atrial cardiopathy and associated with worsened cognitive function.^21^ NT pro-BNP is a serum biomarker released by the myocardium and a predictor of AF, subclinical cerebrovascular disease and stroke, and has been demonstrated strongly associated with left atrial remodeling and dysfunction.^24,25,26,27^ Recent studies have reported a link between increased NT pro-BNP and cognitive decline and dementia.^28^

## METHODS

### Cohort and study design.

**Figure 1:**
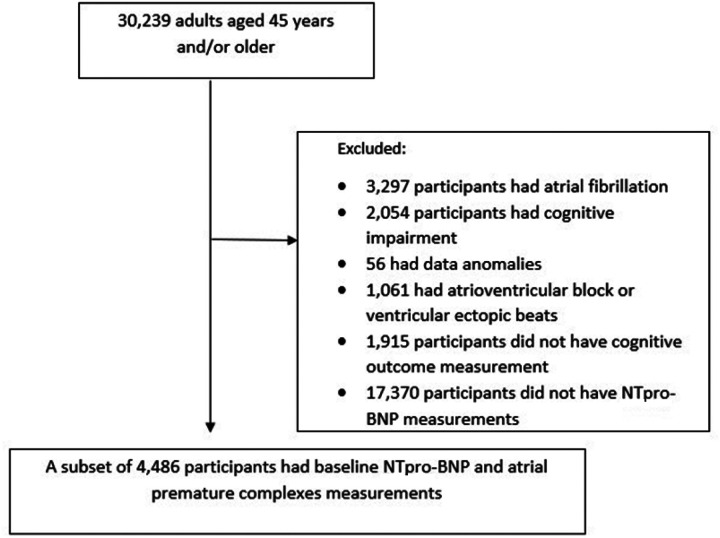
A cohort diagram shows the number of participants included.

The study cohort was derived from the population-based prospective study (Reasons for Geographic and Racial Differences in Stroke, REGARDS). REGARDS enrolled 30,239 adults aged ≥ 45 years between January 2003 and October 2007 and followed them every 6 months by telephone or in-home visits to examine regional and racial differences in stroke mortality.^29,30^ Demographic information and medical histories were obtained via telephone interview and information regarding medications, blood samples, and a resting electrocardiogram (ECG) was collected by trained staff during the in-home visit.^29,30^ The REGARDS data set is available through a data use agreement with the University of Alabama at Birmingham. For the present study, we ascertained a cohort of adults aged ≥ 45 years with measures of atrial premature complexes and (NT pro-BNP) who had no prior AF or cognitive impairment (N = 4,486). Exclusion criteria were a history of AF, cognitive impairment defined as a Six-Item Screener (SIS) score < 5, atherosclerosis, or ventricular ectopic beats. As our focus was on the longitudinal trajectory of cognitive function, those with < 2 cognitive outcome measurements were also excluded (Fig. 1).

### Markers of atrial cardiopathy.

Markers of atrial cardiopathy included atrial premature complexes and NT pro-BNP. Baseline atrial premature complexes were detected on 12-lead ECG and defined by Minnesota code criteria (Minnesota codes 8.1.1, 8.1.3).^31^ For the present analysis, atrial premature complexes were dichotomized as present or absent. NT pro-BNP is a marker of atrial dysfunction and previous research has shown that NT pro-BNP was associated with stroke risk.^32,33^ NT pro-BNP was measured in a random nested case-cohort sample from the REGARDS cohort using an electrochemiluminescence immunoassay.^34^ We treated NT pro-BNP as a continuous variable, and defined elevated NT pro-BNP as those ranked in the top NT pro-BNP quintile.^35^ We also categorized NT pro-BNP into ≤ 54.0, 55.0–124.0, 125.0–299.0, and ≥ 300.0 pg/mL, in accordance with previous literature.^36,37^

### Measures of cognitive function.

Cognitive function was measured using the SIS score, a global measure of cognitive function that can be measured through telephone or face-to-face interview and has previously been shown to be reliable for identifying subjects with cognitive impairment.^38^ Starting in December 2003, participants’ cognitive function was measured annually using the SIS (score range, 0–6).^38^ A score of 5 or 6 was defined as cognitive impairment, and a score of 0–4 was considered normal.^38^ The SIS score has been widely used to identify individuals with cognitive impairment in previous literature, with a sensitivity of 74% and 80% specificity in community setting and 89% and 88% in outpatient setting.^38,39^

### Demographic and clinical characteristics.

In REGARDS, participants reported on their age, sex, race, exercise, smoking status, and alcohol use at baseline. Comorbidities included diabetes, hypertension, hyperlipidemia, left ventricular hypertrophy, and a history of myocardial infarction, stroke and heart diseases. Participants were asked about whether they were ever diagnosed with diabetes (defined as a fasting glucose ≥ 126 mg/dL, a non-fasting glucose ≥ 200 mg/dL, or self-reported diabetes medication use^30,34^), hypertension (defined as systolic blood pressure of 140 mm Hg or more and diastolic blood pressure of 90 mm Hg or more or self-reported antihypertensive medication use^30,34^), and hyperlipidemia (defined as total cholesterol of 240 mg/dL or more, low-density lipoprotein cholesterol of 160 mg/dL or more, high-density lipoprotein cholesterol of 40 mg/dL or less, or self-reported antihyperlipidemic medication use^30,34^). Participants were also asked about whether they had used statin, antiplatelets, or antidepression medications. Myocardial infarction and coronary artery disease were self-reported or diagnosed using ECG data. Left ventricular hypertrophy was measured using ECG data and defined by the Sokolow-Lyon Criteria.^40,41^ A history of stroke was obtained based on clinical review of medical recorded by a team of stroke excerpts, using published guidelines.^30,41^ Data on all of these demographic and clinical characteristics were ascertained for the present study. Incident AF and stroke during the follow-up are considered potential confounders.

### Statistical analysis.

Descriptive statistics were used to characterize the study cohort for demographic and clinical variables. Trajectories of cognitive function over a 8-year follow up were identified using latent class growth modelling, a semiparametric approach that identifies distinct latent subgroups of individuals following a similar pattern of an outcome over time.^42^ The number of trajectory groups was determined by overall model fit as assessed by the Bayesian Information Criterion (BIC), posterior probability, and proportion of individuals in each group.^42^ Trajectories were first specified for one group and additional groups were added until the model fit worsened. We used a censored modelling approach to account for non-random attrition.

Once the number of groups was identified, baseline exposure, demographic and clinical characteristics in each trajectory group, including age, sex, race, exercise, smoking status, alcohol use, prior history of hypertension, diabetes, coronary artery diseases, myocardial infarction, left ventricular hypertrophy, and baseline use of antiplatelet, statin, or anti-depression drugs, were compared. Mean and standard deviation (SD) were used to describe continuous variables, and proportions and percentages were used to describe categorical variables. Analysis of variance was used for continuous variables, and chi-square or Fishers’ exact test was used for categorical data. A multinomial regression model was used to identify independent factors associated with each trajectory group.

In a sensitivity analysis, participants developing incident AF during the study follow-up were removed to estimate the association between atrial cardiopathy and trajectories of cognitive function for adults without incident AF. Similarly, participants with incident stroke during the follow-up were removed to examine the association. Stratified models were also conducted to examine effect modification by sex (males and females).

All analyses were conducted using R software, version 4.0.3 (R Foundation for Statistical Computing, Vienna, Austria).

## RESULTS

### Cohort characteristics.

After applying exclusion criteria as described above, 4,486 participants were included for analysis. Table 1 presents a summary of baseline characteristics. On average, participants were 66.1 years old at baseline (SD = 10.8), with 50.7% females and 56.9% self-identified as white. More than half of the participants were diagnosed with hypertension (62.5%) and dyslipidemia (58.7%) at baseline. A total of 896 participants (20.0%) had elevated NT pro-BNP, and 503 (11.2%) had atrial premature complexes at baseline.

### Trajectories of cognitive function.

**Figure 2:**
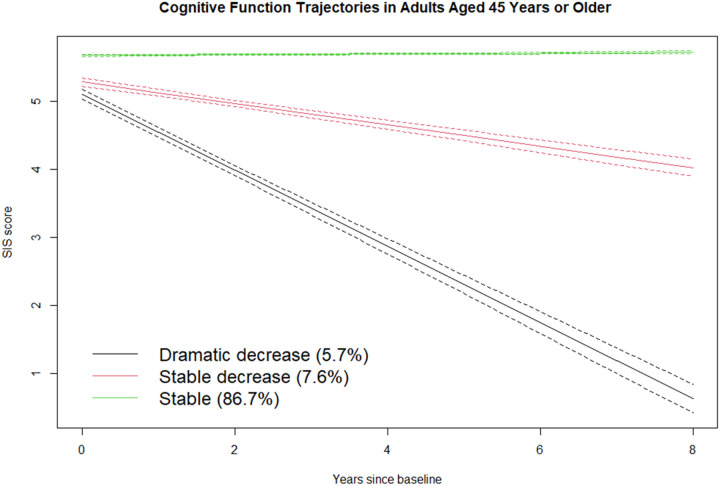
Cognitive trajectories in adults aged 45 years or older. The band around each trajectory represents the 95% confidence interval.

Latent class growth modeling indicated that the data were best modeled as three subgroups of adults with unique cognitive function trajectories (Fig. 2) (BIC: 27574.7). The first group, labeled “stable,” was composed of 86.7% of the participants who had high SIS scores at baseline and remained relatively stable over the follow-up. The second group, “stable decrease,” was composed of 7.6% of the participants who had high SIS scores at baseline and showed a progressively decreasing trajectory. The remaining group, “dramatic decrease,” was composed of 5.7% of the participants who showed a dramatic decrease in SIS score. Table 2 summarizes the SIS scores of each trajectory at each time point. The mean difference between baseline and 8-year follow-up SIS scores was a decrease of 1.6 points (95% confidence interval [CI]: 1.4–1.9; p < 0.01) for participants in the “stable decrease” group and a decrease of 4.1 points (95% CI: 3.6–4.7; p < 0.01) for participants in the “dramatic decrease” group. There was no significant change in SIS score between baseline and 8-year follow-up for participants in the “stable” group.

### Factors associated with each trajectory.

Table 3 summarizes the characteristics of each trajectory. Participants with older age, myocardial infarction, hypertension, elevated NT pro-BNP, and atrial premature complexes were more likely to have a worsening SIS trajectory.

Table 4 summarizes the results of multinomial models and reports adjusted odds ratios (OR) and 95% CI of belonging to each group trajectory relative to the stable group. Older age (OR: 1.08; 95% CI: 1.07–1,11), black participants (white vs black, OR: 0.36; 95% CI: 0.25–0.52), and use of antidepression drugs at baseline (OR: 2.00; 95% CI: 1.22–3.29) were significantly associated with the “dramatic decrease” trajectory. Age (OR: 1.08; 95% CI: 1.06–1.10), black participants (white vs black, OR: 0.37; 95% CI: 0.27–0.52), and male participants (OR: 1.80; 95% CI: 1.30–2.49) were significantly associated with the “stable decrease” trajectory. Elevated NT pro-BNP was significantly associated with the “dramatic decrease” trajectory (OR: 2.14; 95% CI: 1.02–4.49).

Table 5 presents the results of the sensitivity and effect modification analyses. After removing participants who had an incident AF during the follow-up, elevated NT pro-BNP was significantly associated with the “stable decrease” and “dramatic decrease” groups. Removing those who had an incident stroke during the follow-up resulted in insignificant associations between atrial cardiopathy and worse cognitive trajectory. Both elevated NT pro-BNP and atrial premature complexes were significantly associated with the “dramatic decrease” trajectory only in males.

## DISCUSSION

This study identified longitudinal trajectories of cognitive status in adults aged ≥ 45 years and with no prior AF. The longitudinal cohort followed three unique trajectories of cognitive function over time, with 5.7% of adults showing a dramatic decrease in cognitive status. Results of this study suggested that old age and race (black) were significantly associated with a worse trajectory of cognitive function, and elevated NT pro-BNP, a marker of atrial dysfunction,^32,33^ was associated with a decreasing trajectory of cognitive function among those without known AF or incident AF during follow-up. The study also provides novel evidence that among male participants, both elevated NT pro-BNP and atrial premature complexes were significantly associated with increased odds of having a worse cognitive trajectory.

Findings of this study adds to prior evidence on associations between markers of left atrial cardiopathy and cognitive decline independent of AF.^21,43^ Previous cross-sectional analyses of data from the Atherosclerosis Risk in Communities (ARIC) study^21,44^ have demonstrated that a burden of premature atrial contraction is associated with cognitive decline or dementia in community-dwelling older adults without AF, with premature atrial contractions associated with 74% higher odds of prevalent dementia.^21^ Further to this, a retrospective analysis of data from the ARIC study showed that several ECG measures of lower left atrial function (i.e., left atrial reservoir strain, conduit strain, contractile strain, and emptying fraction) were significantly associated with an increased risk of incident dementia in 4,096 older adults without AF or stroke, suggesting that impaired left atrial function is an important risk factor associated with cognitive decline.^22^ NT pro-BNP is a indicator of left atrial dysfunction and has been demonstrated to significantly increase the risk of atrial fibrillation and the odds of dementia independent of vascular risk factors in case-control studies.^32,33,45,46^ The present study extends prior findings in several ways. In this study, cognitive function of a cohort who had measures on atrial cardiopathy markers was assessed annually. With a long follow-up duration, this study allows for examining how markers of atrial cardiopathy are associated with cognitive function over several years. Moreover, this study identified three trajectories of cognitive function in aging adults without AF and showed that atrial cardiopathy markers were associated with a dramatic decline in cognitive status, suggesting that atrial cardiopathy, structural abnormalities and electrical left atrial remodeling preceding clinically AF, may be important determinants of a worse cognitive trajectory.

The observed associations between atrial cardiopathy markers and a worse cognitive trajectory in adults without AF are consistent with existing evidence for a new mechanistic model of atrial cardiopathy, AF and related cognitive outcomes.^47,48^ There has been strong evidence from prospective and retrospective studies that the major pathophysiologic pathway leading from left atrial cardiopathy to the onset of cognitive decline and dementia may not be fully through elevations in thromboembolic risk via the development of clinical AF or ischemic stroke. Instead, atrial cardiopathy, a state of structural abnormality and left atrial remodelling, may be an important impetus for thrombi and subsequent embolism without the development of AF.^49^ Under this new mechanistic model, AF is no longer a necessary step in the pathogenesis of ischemic stroke or cognitive impairment,^47,50^ and potential mechanisms linking left atrial cardiopathy and a worse cognitive trajectory may be microembolism, hypoperfusion, inflammation, breakdown of the blood-brain barrier, and subsequent clinical stroke, similar to mechanisms linking AF and related cognitive outcomes.^48^

The results of this study have implications for the identification of target candidates for preventative interventions to promote cognitive function in ageing population. Although cognitive change is a normal process of ageing, some older adults are more likely to experience more cognitive decline. Analyses of data from the English Longitudinal Study of Ageing showed that gender and age predicted faster decline in all cognitive function domains, including memory, executive function, processing speed and global cognitive function.^51^ Using data from the ASPREE (Aspirin in Reducing Events in the Elderly), another study identified four trajectories of global cognitive function measured using the Mini-Mental State Examination, and the study also found that participants in the lower-functioning trajectory were more likely to be older, male, and with a lower education level.^52^ The present study demonstrated that in addition to demographic factors, markers of left atrial cardiopathy were also associated with a worse cognitive decline, suggesting that ageing adults with evidence of left atrial cardiopathy (i.e., atrial premature complexes and elevated NT pro-BNP) may be a potential therapeutic target for interventions, such as screening services and early treatment of anticoagulation. Notably, the ongoing ARCADIA (Atrial Cardiopathy and Antithrombotic Drugs in Prevention After Cryptogenic Stroke) trial has been testing the efficacy of anticoagulation treatment for stroke prevention in participants with evidence of atrial cardiopathy who did not have AF.^53^ Further studies may examine the efficacy of early preventative services among ageing adults with left atrial cardiopathy.

The results of stratified analyses demonstrated that sex modified the effect of atrial cardiopathy on cognitive function trajectories. For instance, elevated NT pro-BNP and atrial premature complexes were significantly associated with a worse cognitive decline only in males. The findings suggest that different markers may have different relevance for different populations. Further studies may need to explore how other markers of left atrial cardiopathy markers (i.e., left atrial enlargement, P-wave terminal force in lead V1) affect cognitive trajectories across different populations.

This study has several limitations. First, the study only included residents who self-identified as non-Hispanic black or white in the stroke belt region, defined as the 8 southern states (North Carolina, South Carolina, Georgia, Tennessee, Mississippi, Alabama, Louisiana, and Arkansas),^30^ and the stroke mortality rate in this stroke belt region was substantially greater than the national average in the United States.^54^ Thus, the study participants may not be representative of the general population, leading to selection bias. Second, trajectories of cognitive function were modeled based on baseline characteristics. Several important time-varying covariates, such as medication status and comorbidities, were not available for analysis. Third, due to the limited data, this study only considered two markers of atrial cardiopathy (atrial premature complexes and NT pro-BNP). Other markers of atrial cardiopathy, including left atrial volume index, P-wave terminal force at V1, and excessive atrial ectopy, may also be important determinants of cognitive function trajectories.^55,56,57^ Finally, due to the limited data availability, this study only used the SIS score, a brief cognitive function test, to assess cognitive impairment. Although a brief cognitive test can help determine the degree of cognitive status, a diagnosis of cognitive impairment should also be based on other clinical tests such as neurological exams and brain imaging.

In conclusion, atrial premature complexes and elevated NT pro-BNP, both markers of left atrial cardiopathy, are significantly associated with a worse cognitive trajectory in individuals without atrial fibrillation. These findings have implications for the early identification of high-risk populations and the development of prevention services to promote cognitive health in ageing populations.

## Figures and Tables

**Table 1:** Baseline characteristics of the cohort (N = 4486).

Baseline characteristics	Mean (SD), or N (proportion)
Age, mean	66.1 (10.8)
Gender, female	2274 (50.7%)
Race, white	2554 (56.9%)
Exercise	
1–3 times per week	1571 (35.0%)
4 or more time per week	1319 (29.4%)
Smoke, current	695 (15.5%)
Smoke, past	1739 (38.8%)
Alcohol, current	2220 (49.5%)
Alcohol, past	865 (19.3%)
Myocardial infarction	551 (12.3%)
Coronary artery disease	781 (17.4%)
Diabetes	1093 (24.4%)
Hypertension	2802 (62.5%)
Dyslipidemia	2633 (58.7%)
Stroke	149 (3.3%)
Medication use, statin	1408 (31.4%)
Medication use, antiplatelets	240 (5.3%)
Medication use, antidepression	532 (11.9%)
Medication use, diabetes	921 (20.5%)
Left ventricular hypertrophy	152 (3.4%)
Elevated NT pro-BNP (top quintile)	896 (20.0%)
NT pro-BNP, ≤ 54.0 pg/mL	1503 (33.5%)
NT pro-BNP, 55.0–124.0 pg/mL	1073 (23.9%)
NT pro-BNP, 125.0–299.0 pg/mL	800 (17.8%)
NT pro-BNP, ≥ 300.0 pg/mL	729 (16.3%)
Atrial premature complexes	503 (11.2%)

N, number of participants. SD, standard deviation. NT pro-BNP, N-Terminal pro-Brain Natriuretic Peptide.

**Table 2:** SIS score of each trajectory at each time point

SIS score at baseline and during follow-up	Group 1	Group 2	Group 3
	Stable	Stable decrease	Dramatic decrease
Baseline	5.8 (0.4)	5.5 (0.5)	5.5 (0.5)
1 year	5.5 (0.7)	4.7 (1.0)	4.1 (1.4)
2 years	5.7 (0.6)	4.9 (1.0)	3.8 (1.5)
5 years	5.7 (0.6)	4.5 (1.0)	2.3 (1.5)
8 years	5.8 (0.5)	3.9 (1.4)	1.2 (1.1)
Change in SIS score (95% CI)	0.0	−1.6	−4.1
	(−0.02, 0.03), p = 0.94	(−1.9, −1.4), p < 0.01	(−4.7, −3.6), p < 0.01

SIS, Six-Item Screener. CI, confidence interval.

**Table 3:** Characteristics of three cognitive trajectories.

	Group 1, Stable (N = 3891)	Group 2, Stable decrease (N = 341)	Group 3, Dramatic decrease (N = 254)	p-value
Age, mean	65.0 ± 10.6	72.4 ± 9.5	74.7 ± 9.7	< 0.01
Gender, female	2004 (51.5)	135 (39.6)	135 (53.1)	< 0.01
Race, white	2312 (59.4)	139 (40.7)	103 (40.6)	< 0.01
Exercise				
1–3 times per week	1385 (35.6)	118 (34.6)	68 (26.8)	0.02
4 or more time per week	1154 (29.7)	90 (26.4)	75 (29.5)	0.45
Smoke, current	626 (16.1)	38 (11.1)	31 (12.2)	0.02
Smoke, past	1502 (38.6)	145 (42.5)	92 (36.2)	0.25
Alcohol, current	1974 (50.7)	146 (42.8)	100 (39.4)	< 0.01
Alcohol, past	736 (18.9)	79 (23.2)	50 (19.7)	0.16
Myocardial infarction	453 (11.6)	55 (16.1)	43 (16.9)	< 0.01
Coronary artery disease	647 (16.6)	80 (23.5)	54 (21.3)	< 0.01
Diabetes	925 (23.8)	95 (27.9)	73 (28.7)	0.06
Hypertension	2385 (61.3)	237 (69.5)	180 (70.9)	< 0.01
Dyslipidemia	2295 (59.0)	185 (54.3)	153 (60.2)	0.21
Stroke	12 (0.003)	114 (33.4)	23 (9.1)	< 0.01
Medication use, statin	1212 (31.1)	110 (32.3)	86 (33.9)	0.62
Medication use, antiplatelets	193 (4.9)	28 (8.2)	19 (7.5)	0.01
Medication use, antidepression	459 (11.8)	36 (10.6)	37 (14.6)	0.31
Medication use, diabetes	781 (20.0)	85 (24.9)	55 (21.7)	0.09
Left ventricular hypertrophy	125 (3.2)	16 (4.7)	11 (4.3)	0.24
NT pro BNP quintile, 1st	834 (21.4)	45 (13.2)	20 (7.9)	< 0.01
NT pro BNP quintile, 2nd	797 (20.5)	62 (18.2)	39 (15.4)	0.10
NT pro BNP quintile, 3rd	784 (20.1)	70 (20.5)	45 (17.7)	0.63
NT pro BNP quintile, 4th	754 (19.4)	76 (22.3)	64 (25.2)	0.04
NT pro BNP quintile, 5th	722 (18.6)	88 (25.8)	86 (33.9)	< 0.01
NT pro-BNP, ≥300.0 pg/mL	272 (7.0%)	30 (8.8%)	33 (13.0%)	< 0.01
Atrial premature complexes	403 (10.4)	51 (15.0)	49 (19.3)	< 0.01

N, number of participants. NT pro-BNP, N-Terminal pro-Brain Natriuretic Peptide.

**Table 4:** Adjusted odds ratios and 95% confidence interval of belonging to each group trajectory relative to the stable group

Baseline characteristics	Class 2: Stable decrease	Class 3: Dramatic decrease
Age	1.08 (1.06–1.10)	1.08 (1.07–1.11)
Race – white vs. black	0.37 (0.27–0.52)	0.36 (0.25–0.52)
Males	1.80 (1.30–2.49)	0.87 (0.59–1.28)
Exercise		
1–3 times/week	1.22 (0.85–1.74)	1.08 (0.71–1.65)
4 + times/week	1.05 (0.71–1.54)	1.34 (0.88–2.07)
Smoke, current	0.71 (0.42–1.20)	0.99 (0.56–1.77)
Smoke, past	0.84 (0.60–1.19)	0.88 (0.59–1.32)
Alcohol, current	0.86 (0.59–1.23)	0.87 (0.58–1.30)
Alcohol, past	1.13 (0.74–1.73)	0.70 (0.41–1.19)
Myocardial infarction	0.75 (0.39–1.45)	1.73 (0.68–4.42)
Coronary artery disease	1.65 (0.90–3.00)	0.74 (0.30–1.81)
Diabetes	0.91 (0.48–1.71)	1.46 (0.75–2.83)
Hypertension	0.93 (0.67–1.29)	0.97 (0.65–1.44)
Dyslipidemia	0.76 (0.54–1.07)	1.20 (0.81–1.78)
Stroke	1.05 (0.38–2.87)	1.54 (0.50–4.79)
Baseline use of medications		
Statin	0.82 (0.56–1.19)	0.80 (0.52–1.22)
Diabetes	1.26 (0.65–2.44)	0.73 (0.36–1.49)
Antiplatelets	1.39 (0.79–2.47)	0.95 (0.46–1.97)
Antidepression	1.41 (0.88–2.26)	2.00 (1.22–3.29)
Left ventricular hypertrophy	1.36 (0.76–2.46)	1.02 (0.52–2.03)
Elevated NT pro BNP (top quintile)	1.03 (0.58–1.83)	2.14 (1.02–4.49)
NT pro-BNP, ≤ 54.0 pg/mL (reference)		
NT pro-BNP, 55.0–124.0 pg/mL	1.04 (0.64–1.69)	1.00 (0.53–1.91)
NT pro-BNP, 125.0–299.0 pg/mL	1.21 (0.72–2.06)	0.94 (0.47–1.88)
NT pro-BNP, ≥ 300.0 pg/mL	0.79 (0.41–1.52)	0.95 (0.44–2.04)
Atrial premature complexes	1.01 (0.66–1.55)	1.18 (0.73–1.89)

NT pro-BNP, N-Terminal pro-Brain Natriuretic Peptide.

**Table 5:** Sensitivity and effect modification analyses.

	Class 2: Stable decrease	Class 3: Dramatic decrease
Participant without incident AF		
Elevated NT pro-BNP (top quintile)	2.50 (1.22–5.11)	11.2 (1.12–112.10)
Atrial premature complexes	1.49 (0.85–2.61)	1.15 (0.31–4.28)
Participant without incident stroke		
Elevated NT pro-BNP (top quintile)	0.72 (0.02–28.45)	0.23 (0.10–8.77)
Atrial premature complexes	1.20 (0.10–14.58)	0.87 (0.07–10.06)
Females		
Elevated NT pro-BNP (top quintile)	0.84 (0.62–1.13)	0.95 (0.68–1.32)
Atrial premature complexes	1.05 (0.83–1.35)	1.19 (0.93–1.52)
Males		
Elevated NT pro-BNP (top quintile)	1.43 (0.99–2.04)	3.88 (1.95–7.72)
Atrial premature complexes	1.02 (0.80–1.31)	1.74 (1.23–2.48)

AF, atrial fibrillation. NT pro-BNP, N-Terminal pro-Brain Natriuretic Peptide.

## Data Availability

In order to abide by its obligations with NIH/NINDS and the Institutional Review Board of the University of Alabama at Birmingham, REGARDS facilitates data sharing through formal data use agreements. Any investigator is welcome to access the REGARDS data through this process. Requests for data access may be sent to regardsadmin@uab.edu.

## Abbreviations

AF: atrial fibrillation
APCs: atrial premature complexes
NT-proBNP: N-Terminal pro-Brain Natriuretic Peptide
SIS: Six-Item Screener
REGARDS: REasons for Geographical and Racial Differences in Stroke
HR: hazard ratio
CI: confidence interval
